# Quantifying the Burden of Rhodesiense Sleeping Sickness in Urambo District, Tanzania

**DOI:** 10.1371/journal.pntd.0000868

**Published:** 2010-11-02

**Authors:** Lucas E. Matemba, Eric M. Fèvre, Stafford N. Kibona, Kim Picozzi, Sarah Cleaveland, Alexandra P. Shaw, Susan C. Welburn

**Affiliations:** 1 Tabora Research Centre, National Institute for Medical Research, Tabora, Tanzania; 2 Centre for Infectious Diseases, School of Biomedical Sciences, College of Medicine and Veterinary Medicine, University of Edinburgh, Edinburgh, United Kingdom; 3 Ashworth Laboratories, Centre for Infectious Diseases, School of Biological Sciences, College of Science and Engineering, University of Edinburgh, Edinburgh, United Kingdom; 4 Division of Ecology and Evolutionary Biology, University of Glasgow, Glasgow, United Kingdom; 5 AP Consultants, Abbotts Ann, Andover, United Kingdom; Foundation for Innovative New Diagnostics (FIND), Switzerland

## Abstract

**Background:**

Human African trypanosomiasis is a severely neglected vector-borne disease that is always fatal if untreated. In Tanzania it is highly focalised and of major socio-economic and public health importance in affected communities.

**Objectives:**

This study aimed to estimate the public health burden of *rhodesiense* HAT in terms of DALYs and financial costs in a highly disease endemic area of Tanzania using hospital records.

**Materials and Methods:**

Data was obtained from 143 patients admitted in 2004 for treatment for HAT at Kaliua Health Centre, Urambo District. The direct medical and other indirect costs incurred by individual patients and by the health services were calculated. DALYs were estimated using methods recommended by the Global Burden of Disease Project as well as those used in previous *rhodesiense* HAT estimates assuming HAT under reporting of 45%, a figure specific for Tanzania.

**Results:**

The DALY estimate for HAT in Urambo District with and without age-weighting were 215.7 (95% CI: 155.3–287.5) and 281.6 (95% CI: 209.1–362.6) respectively. When 45% under-reporting was included, the results were 622.5 (95% CI: 155.3–1098.9) and 978.9 (95% CI: 201.1–1870.8) respectively. The costs of treating 143 patients in terms of admission costs, diagnosis, hospitalization and sleeping sickness drugs were estimated at US$ 15,514, of which patients themselves paid US$ 3,673 and the health services US$ 11,841. The burden in terms of indirect non-medical costs for the 143 patients was estimated at US$ 9,781.

**Conclusions:**

This study shows that HAT imposes a considerable burden on affected rural communities in Tanzania and stresses the urgent need for location- and disease-specific burden estimates tailored to particular rural settings in countries like Tanzania where a considerable number of infectious diseases are prevalent and, due to their focal nature, are often concentrated in certain locations where they impose an especially high burden.

## Author Summary

Sleeping sickness (human African trypanosomiasis - HAT) is a disease transmitted by tsetse flies and is always fatal if left untreated. The disease occurs in foci affecting poor communities with limited access to health service provision and as such the disease is often left undiagnosed, mistaken for more common afflictions. Even if diagnosed, sleeping sickness is costly to treat, both for health services and patients and their families in terms of costs of diagnosis, transport, hospital care, and the prolonged period of convalescence. Here we estimate the health burden of the “acute form” *T. b. rhodesiense* sleeping sickness in Urambo District, Tanzania in terms of Disability Adjusted Life Years (DALYs), the yardstick commonly used by policy makers to prioritize disease management practices, representing a year of healthy life lost to disease. In this single district, the burden of the disease over one year was estimated at 979 DALYs and the estimated monetary costs to health services for the 143 treated patients at US$ 11,841 and to the patients themselves at US$ 3,673 for direct medical costs and US$ 9,781 for indirect non-medical costs. Sleeping sickness thus places a considerable burden on the affected rural communities and health services.

## Introduction

Sustainable solutions to most of the neglected tropical infections, which include human African trypanosomiasis (HAT) [1], remain illusory in most of the poor rural communities living in third world countries. Whilst significant, but still insufficient, recourses have been directed towards research and control of those diseases which are capable of making sizeable contributions to global pandemics such as HIV, Avian influenza, H5N1 and most recently H1NI, political bias and a myriad of other factors have led woefully inadequate resources being allocated to dealing with less high profile diseases [2]. Poor countries are thus left alone struggling to control a handful of endemic ‘neglected’ infectious diseases [3]. Many of these diseases occur in clusters within the same individuals the so-called “polyparasitic” [3], [4] and often alongside other conditions, including the major killer diseases [5]. Frustratingly, amongst the neglected tropical diseases, some diseases are more neglected than others both locally and internationally. Furthermore, many neglected disease are also zoonotic [6], affecting both humans and animals and imposing a dual burden on human and animal health. As such these diseases have a major impact on rural livelihoods by contributing towards the increasing the level of poverty in most of these communities, which are already poor. Sleeping sickness or human African trypanosomiasis (HAT) is a classic example of a neglected disease. The ‘acute’ form of HAT found in eastern Africa and caused by *Trypanosoma brucei rhodesiense* is zoonotic and needs to be addressed from human and animal perspectives. Some authors [7], [8] have notably argued that the burden of the *rhodesiense* form of HAT can be significantly reduced by treating domestic livestock reservoirs as this can play an important role in controlling HAT in humans. This has significant cost implications for rural medical and veterinary services.

The public health burden for HAT has been estimated at 1,609,000 DALYs with 50,000 annual deaths [9], although there are many methodological and data issues that deserve greater consideration when making such estimates for HAT [10]. The HAT figure none-the-less seems small when compared with the standard (discounted at 3% and age-weighted) DALY burden in Africa of (46.7 million due to HIV/AIDS (Human immunodeficiency virus/Acquired Immune Deficiency Syndrome), 30.9 million from malaria and 10.8 million from tuberculosis [8]. However, HAT, like many other neglected diseases, is highly focalized, and its burden therefore needs to be considered in relation to the affected localities. There has been a dramatic reduction in the number of reported HAT cases, which declined to 15 and to 17,000 cases per year [11] in the mid-2000s from nearly 40,000 at the end of the 1990s, but major challenges still exist in predicting future disease trends. With only six years remaining to the year targeted by World Health Organization (WHO) for HAT elimination, the disease situation is still unclear in about one third of the countries where it is endemic [11]. Accurate estimates of incidence and effective control of HAT, especially of the chronic West and Central African form caused by *T. b. gambiense*, depend on active surveillance which is expensive and requires a high level of organization. Among vector-borne diseases in Africa, HAT ranks second for mortality and fourth in terms of disability adjusted life years (DALYs), but the fact that this is a severely under-reported disease [12] has largely prevented accurate assessment of its true burden.

In the United Republic of Tanzania, HAT is a disease of major public health and socio-economic importance in affected rural communities, where it continues to impose a serious threat to the 4–5 million people exposed to it. Between 1996 and 2006, 2748 cases were reported in Tanzania [11], [13]. This figure represents an average of about 250 cases of *T. b. rhodesiense* reported annually, and over 40% of the global total. More than 95% of the cases were reported from just three regions in the western part of the country. Tanzania still has several active HAT foci which have been persistent for over 80 years. The disease, which was effectively controlled in early 1960s, made a dramatic re-emergence in some parts of the country during the 1990s [14], [15], due to a lack of adequate and sustained control activities. Here we attempt to quantify the burden of zoonotic HAT imposed in rural Tanzania in recent years.

## Materials and Methods

Between 2000 and 2007, five rural districts of Tanzania, Kigoma, Kasulu, Kibondo, Urambo and Mpanda, all located in the western part of the country, reported cases of sleeping sickness. Out of the five districts, Urambo was purposefully selected for two main reasons; first it recorded the highest number of cases, and second it had the best record-keeping for HAT. Thus, our study relates to the burden of HAT in the high incidence district of Urambo.

### Study area and population

**Figure 1:**
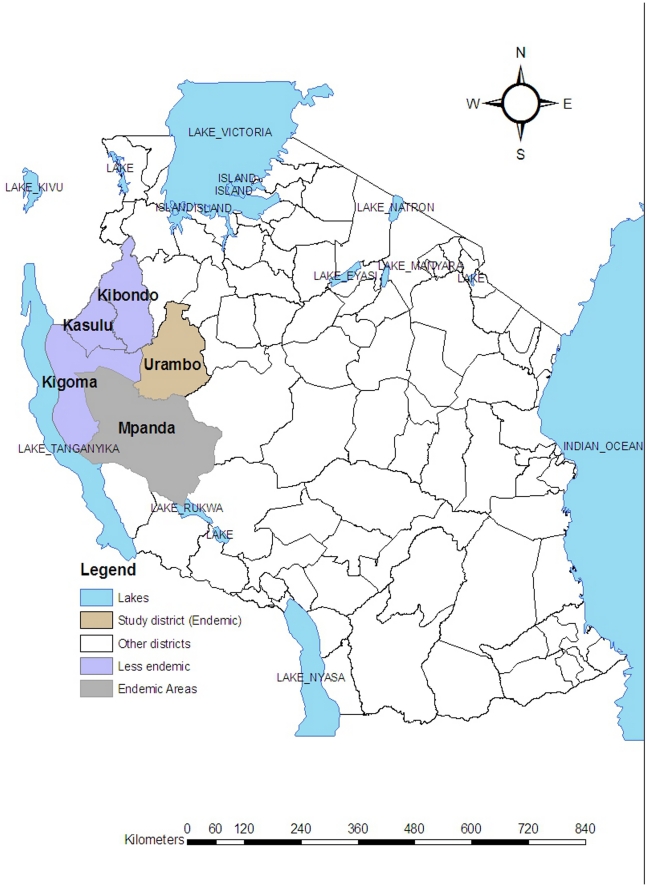
Map of Tanzania showing the location of districts affected by sleeping sickness including Urambo district.

Urambo District (Figure 1) is situated in Tabora region, which is in the western part of the country between latitude 4° 00″–5° 53″S and longitude 30° 00′–32° 37″ E. It occupies 25,995 km^2^ with a total population of 369,329 in 2002 [16]. The district shares a common border with Mpanda, Kigoma rural and Kasulu districts where HAT is also endemic. The predominant ethnic groups are Nyamwezi and Sukuma with some Fipas. Kinyamwezi is the most widely spoken language, although Swahili, the national language, is also used. Subsistence farming is widely practiced, the main crops being tobacco, rice and maize.

### Data collection

All the patients included in the present study were diagnosed and treated in Kaliua Health Centre. Three cases were diagnosed during active surveillance activities carried out in Urambo district, which were also referred to Kaliua for treatment. The remaining patients presented passively. Kaliua is a missionary health facility that was selected by the district health authorities to manage all HAT cases in Urambo district, due to its location. It is within the disease catchment area and is easy accessible by train to patients from all affected villages. Information about HAT patients diagnosed and treated in Kaliua between 2000 and 2007 was obtained from hospital registers. Patient records were categorized by sex, age cohort, stage of the disease, dates of admission and discharge, initial and final diagnosis; treatment provided (this was done with the intention of confirming the stage of the disease), duration of hospitalization and finally the outcome of the disease. The distinction between first/early stage patients (where the parasite is circulating in the blood and lymph system) and second/late stage (where it has passed through the blood brain barrier into the cerebro-spinal fluid) determines what treatment patients are given. For *T. b. rhodesiense*, first stage patients receive Suramin and second stage patients Melarsoprol [17]. It was not possible to obtain disease stage data for 22 of the patients (as this was not recorded by the care team and further records were unavailable). However, all these un-staged patients recovered and were discharged from the hospital. Therefore, using the conservative assumption that there are fewer non drug-related complications and there is no increased non drug-related mortality in the early stage of the disease, the 22 patients were assumed to be early stage.

### Data analysis

Population, incidences and number of deaths were grouped into 5 year age cohorts for ages 5 to 79, and into <1 year, 1–4 years, and 80+ years. Data were manipulated in Microsoft Excel 2003 and analysed in a fully stochastic framework run using the @Risk software package (Palisade, Newfield, NY, USA version 5.0), as described in [7]. The methodology captures both uncertainty and annual variability in the estimates. Both life expectancy data and the template for the DALY calculation were obtained from WHO website (http:/www.who.int/evidence). The life table used in this study was Tanzania-specific [8].

The DALYs calculations for HAT in Tanzania used an adapted stochastic approach [8]; 10,000 Monte-Carlo simulations were run using, at each iteration, input values drawn from the data of the same year per iteration. Final output data was provided as means with 95% confidence intervals.

### Disability Adjusted Life Years (DALYs) for *T. b. rhodesiense* HAT

The total DALY score for each cause-age-sex group were calculated as the sum of non-fatal burden (years of life lived with disability - YLD) and the burden of premature mortality (years of life lost - YLL). To calculate the DALY score for *T. b. rhodesiense* HAT in Urambo, we applied a 3% discount rate, both with and without age-weighting. The information required for DALY estimations is shown in Table 1. The population data for Urambo District used in the current calculations was projected for the year 2004 from the 2002 Tanzania population and housing census data by applying the estimated annual population growth rate of 4.8% per annum [16] using the standard compound growth rate formula to obtain an estimate of 406,235.

**Table 1:** Information required for the estimation of disability adjusted life years (DALYs) for *rhodesiense* sleeping sickness.

DALY component	Type of data required	Source of data used in this study
Years of Life Lost (YLL)	1) Number of deaths	Health facility records of case fatality, estimates of under-reporting.
	2) Life expectancy at birth	Life tables obtained from WHO website, based on African standard life tables specific for Tanzania for the year 2004, with life expectancy at birth of 48.0 years which was assumed to be representative for the population of Urambo district for the year 2004.
	3) Distribution of deaths according to age	Health facility records, summarized for each age group (with and without age weighted case fatality rate and under-reporting rate) using methods developed for Uganda [12].
Years Lost due to Disability (YLD)	4) Disability weighting	From *rhodesiense* sleeping sickness records as per Murray (1994) methods and also using expert opinion.
	5) Duration of illness	Health facility records excluding post treatment duration.
	6) Age weighting	Health facility records of productivity of different age groups.

### Years of Life Lost (YLL)

To calculate YLL for Urambo, standard global burden of disease (GBD) methods were followed, using the age categories described above. All other information required for the calculation is shown in Table 1. The total number of reported deaths and estimated under-reporting for Tanzania are described elsewhere [18]; we used under-reporting rates of 0% (no under-reporting), and the figure 45% under-reporting (that is for every 100 reported cases, a further 45 remained un-reported), which had already been derived [16].

### Years Lost due to Disability (YLD)

As previously published [8], we use a disability weighting of 0.21 for early stage HAT and 0.81 for late stage episodes based on standard definitions [19]. Previous DALY estimates for *T. b. rhodesiense* used a weighting of 0.35 (originally devised for *T. b. gambiense* infection); we also ran our model with this value, for comparison. Although long term effects, sequelae, from HAT are known to exist [20] the data to support a calculation of these for this sample did not exist and therefore sequelae were not included.

### Estimating Disability Adjusted Life Years in the absence of the HIV/AIDS pandemic

AIDS has a significant impact on burden of disease estimates. It is estimated that life expectancy for Tanzanians fell from 54 years in 1990 to 45.9 years in 2004 [21] and that the incidence of AIDS will reduce life expectancy in Tanzania by 17% during 2000–05, by 14% during 2010–2015 and by 7% during 2045–50 [22]. The disease that has already increased mortality by 11 percent has also resulted in welfare losses equivalent to 47.2 percent of GDP [23]. In realizing this importance, if we assume that HIV pandemic did not occur, then the burden due to most of diseases would probably have been different from what we observe today. In an attempt to capture and compare this component of calculations, we decided to recalculate DALYs using the same set of data but with the assumption that HIV pandemic did not occur. We therefore re-calculated the DALY score using the life expectancy of Tanzanians for the year 1990, when HIV infection was not very severe in the country.

### Estimating the direct costs of hospitalisation at the health facility

The costs to the health system per HAT patient were estimated from four components i) the product of the total recorded hospital stays due to HAT in days multiplied by an estimated daily cost for hospital services plus ii) the estimated cost of diagnosing HAT patients plus iii) a value for the drugs used to treat HAT less iv) the amounts that patients paid towards these costs. The total number of days of hospitalisation due to HAT in 2004 at the Kaliua Health Centre was 3601, or a little more than 25 days per parasitologically confirmed HAT patient. Kaliua Health Centre being a missionary hospital, patients are only charged a nominal amount of Tanzanian Shillings (TZS) 1000 (approximately US$ 1), per every patient per night bed occupancy [24], plus a one off payment of TZS 500 (approximately US$ 0.5) for initial laboratory tests on admission. The true cost of these tests would be higher. Here we assume that, in line with the figures cited in [17] the total cost would be at least US$1. This hospitalisation charge is also low, compared, for example with costs for Uganda [7] which were estimated at US$ 2 per day's hospitalisation for HAT, in itself a low figure. The US$ 2 figure was retained as an estimate of the true cost of hospitalisation, half of which was covered by the patients' nominal fee. Thus we conservatively assumed that the fees charged to patients for hospitalisation and admission covered half the actual cost to the health services. Charges for other services such as additional laboratory investigations and treatment incurred by individual patients (depending on the clinical presentation and severity of the condition) were not included in this part of the study. The local currency was converted using the 2004 rates taken from the Bank of Tanzania, during which US$ 1was equivalent to TZS 1000.

Turning to drugs, all the drugs used to treat HAT cases in Tanzania are provided free of charge by WHO. Drugs are bought by WHO from the manufacturers at a greatly subsidised price. The Tanzanian Ministry of Health and Social Welfare is then responsible for making these drugs available to all treatment centres. Finding an appropriate value for the drugs is thus complex, so the compromise of using the costs at the levels paid by WHO is used here. Accordingly the costs for the 30 early and 113 late stage cases were estimated and added to the costs, using the rate of US$ 35 for early and US$ 63 for late stage estimated by WHO.

Other additional costs were difficult to quantify and were excluded from the current estimates since they vary greatly according to each individual presentation. These include drugs to treat individual presentations such as fever, anaemia, pain, adverse drug events and all concomitant conditions. However, transport to and from the hospital, living costs during hospital stay and costs to cover living expenses for one accompanying person were regarded as patients' non-medical or indirect costs and were estimated. All HAT patients require assistance with daily living activities, such as meal preparation, shopping and housework.

### Costs borne by patients

The costs borne by patients were firstly, the direct medical costs in terms of the US$ 1 fee per day's hospital stay and the US$ 0.50 admission fee and secondly indirect, non-medical costs. These were estimated in terms of transportation and living costs incurred by each patient during the entire duration of hospitalization. Data used in this part of study were provided by relatives of the patients who were admitted at Kaliua Health Centre during the course of the study and also patients who recovered from HAT who were followed up in their homes in a separate study conducted towards the end of 2007. Travel costs were estimated using reasonable rail fares (roads in most of the disease endemic villages are impassable during the rainy season and the only reliable means of transport is through the railway system; all HAT-affected villages were accessible by train).

The village of origin for all 143 patients were obtained from the hospital register. Standard travel costs were estimated based on the information provided by both patient and relatives, the majority reported that they travelled on the third class coach; therefore third class fare information for all destinations was obtained from the station master at Kaliua railway station. The basis for the estimates is shown in Table 2. The 143 patients spent a total of US$ 479.50 on transport from their home village to Kaliua Health Centre. This is equivalent to US$ 3.35 per patient per single trip to Kaliua, or US$ 6.70 for a return trip. HAT patients also require assistance with food preparation and personal care, meaning that each patient required one accompanying adult person (over 18 years) for TZS 600 per day to buy meals for their relative from a local restaurant. Since the majority of patients were from villages located far from Kaliua we estimated this using the modest costs of ordering food from a nearby restaurant. We assumed that the accompanying person spent the same amount per day on meals.

**Table 2:** Non-medical indirect costs incurred per individual sleeping sickness patient attending Kaliua health centre estimated in US$.

	Patient US$	Accompanying person US$	Total per patient US$
**Travel**	6.7	6.7	13.4
**Meal**	15.0	15.0	30.0
**Accommodation**	0.0	25.0	25.0
**Total**	21.7	46.7	68.4

The living costs for an accompanying person were estimated at the rate TZS 500 per person per night spent in a local guesthouse (US$ 0.50). All other expenses such as costs required for general care and personal hygiene or any other basic needs for the patient were not included in the current estimates as they varied from one patient to another.

### Ethical statement

Permission to conduct this study in Tanzania was obtained from the Research Ethics Sub-Committee of the National Institute for Medical Research as well as the District Health Authorities of United Republic of Tanzania.

## Results

**Figure 2:**
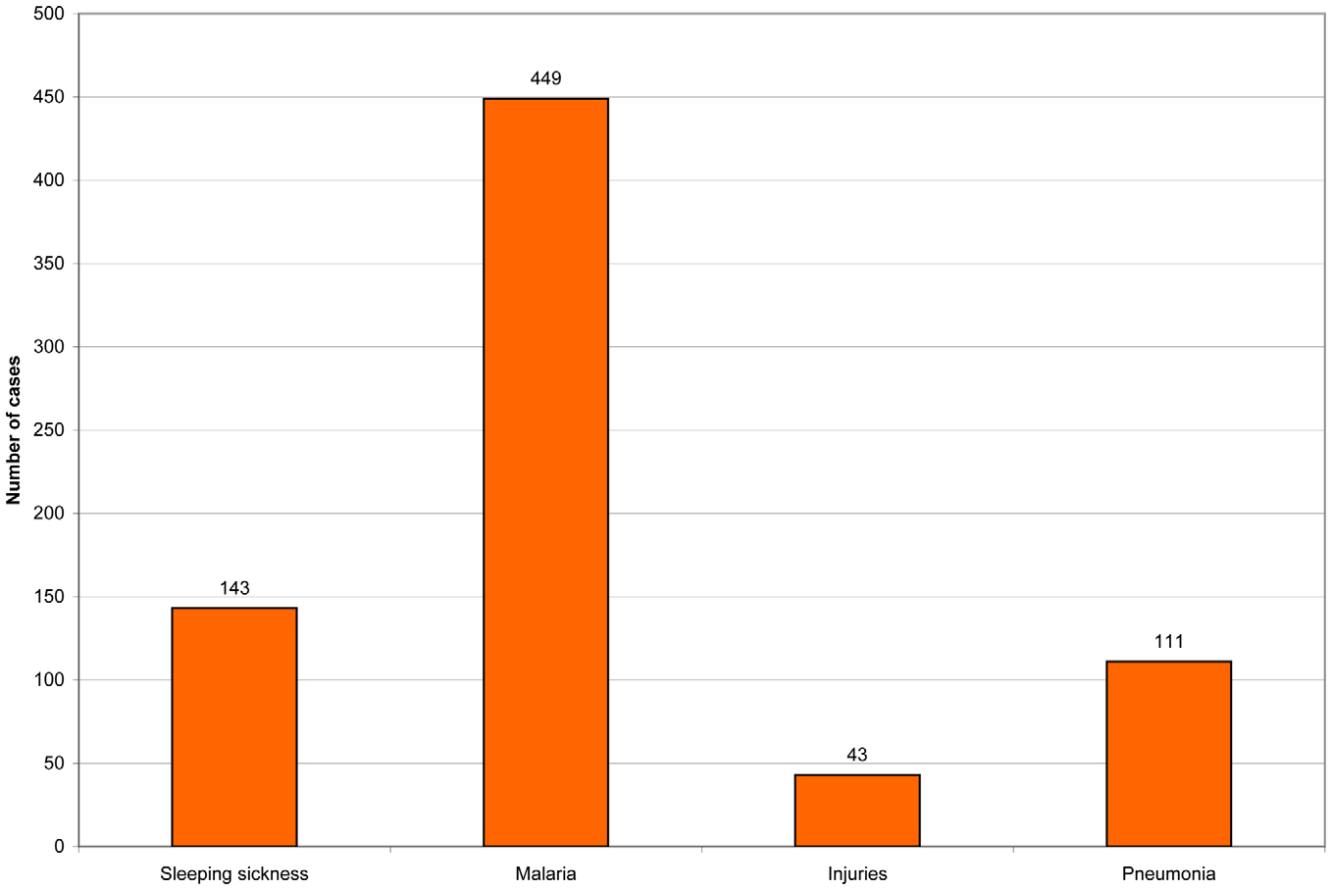
Recorded incidence of selected health conditions at Kaliua Health Centre from January to December 2004.

Between 2000 to 2007, a total of 521 HAT cases were diagnosed and treated at Kaliua Health Centre as follows; 29 patients were diagnosed in 2000, 38 cases in 2001, 58 cases in 2002, 98 cases in 2003, 143 cases in 2004, 83 in 2005, 33 in 2006 and 39 cases in 2007. The highest number of cases of HAT was observed in the year 2004, and this year was selected for the study. As an example, some of the other conditions diagnosed at Kaliua for the year 2004 are shown in Figure 2.

### *T. b. rhodesiense* HAT Disability Adjusted Life Years (DALYs)

The overall DALYs burden due to *T. b. rhodesiense* HAT in Urambo is the sum of all the YLLs and YLDs estimated from the model using annual age specific data for both reported and estimated (at 0.45 (95% CI: 0.36–0.53) unreported deaths, and non age weighted discounted YLL (DYLL) as shown in Table 3. The model also estimated that premature mortality due to HAT was responsible for 190.1 (95% CI: 17.5–250.0) years of life if we assume that all HAT cases were reported. When 45% under-reporting with age weighting and without age weighting are taken into account then HAT was responsible for 1030.5 (95% CI: 201.9–1747.3) and 610.8 (95% CI: 17.5–938.4) YLLs respectively. Table 3 shows the results of years of life lost for each age group. The model also estimated that *T. b. rhodesiense* HAT was responsible for 22.0 (95% CI: 0–63.8) years of life lived with disability in Urambo district, when no age weighting and no under-reporting was applied. When age weighting was added then this result increases to 25.5 (95% CI: 0–67.4) YLDs. A full breakdown of age specific years lived with disability in Urambo district is shown in Table 4.

**Table 3:** Years of Life Lost (YLL) due to *T.b. rhodesiense* sleeping sickness in Urambo, Tanzania.

Age of onset (years)	Total population	Total reported cases	Total reported deaths	Total un-detected deaths with 45% under-reporting	YLLs with 0% under-reporting and no age weighting	YLLs with 0% under-reporting and with age weighting	YLLs with 45% under-reporting and no age weighting	YLLs with 45% under-reporting and with age weighting
0–4	14,547	5	0	2.25 (1.8–2.7)	0	0	27.7 (0–75.1)	33.8 (0–90.9)
5–14	63,082	15	1	6.75 (5.4–8.1)	147.5	202	166.1 (147.5–209.1)	226.8 (202.0–284.1)
15–29	104,073	49	3	22.1 (17.6–26.5)	31.6 (0–64)	41.9 (0–85.3)	208.8 (0–399.6)	281.9 (0–541.8)
30–44	26,376	34	1	15.3 (12.2–18.4)	9.1 (0–19.5)	10.4 (0–23.8)	83.0 (0–196.5)	96.5 (0–228.5)
45–59	53,346	23	1	10.4 (8.3–12.4)	3.6 (0–14.9)	3.4 (0–14.6)	49.1 (0–105.5)	43.4 (0–91.2)
60–69	11,240	9	1	4.1 (3.2–4.9)	1.1 (0–8.5)	0.7 (0–5.6)	21.4 (0–42.1)	15.3 (0–30.8)
70–79	6711	5	0	2.25 (1.8–2.7)	0	0	6.0 (0–14.6)	3.5 (0–8.7)
80+	3542	3	0	1.4 (1.1–1.62)	0	0	2.3 (0–7.0)	1.1 (0–3.3)
**Total**	**405,636**	**143**	**7**	**64.4 (51.5–77.2)**	**192.9 (147.5–250.0)**	**258.3 (202.0–322.5)**	**564.4 (147.5–966.4)**	**702.4 (202.0–1200.3)**

All values are means with lower and upper 95% confidence intervals in brackets as obtained from 10,000 Monte-Carlo simulations derived from the 2000–2007 Urambo HAT data.

**Table 4:** Years of Life Lived with Disability (YLD) due to *T.b. rhodesiense* HAT in Urambo.

Age of onset (years)	Total population	Total reported cases	YLD for recovered cases (early and late stage) and no age weighting	YLD for recovered cases (early and late stage) and with age weighting
0–4	14,547	5	0.8 (0.7–0.8)	0.3
5–14	63,082	15	2.8 (0.2–6.7)	3.1 (0.3–7.5)
15–29	104,073	49	2.4 (0.2–4.8)	3.6 (0.2–7.3)
30–44	26,376	34	7.0 (1.8–14.8)	9.6 (2.5–20.4)
45–59	53,346	23	8.4 (1.6–16.9)	8.9 (1.8–18.0)
60–69	11,240	9	7.1 (2.1–14.0)	5.7 (1.6–11.2)
70–79	6711	5	6.8 (0–24.0)	4.2 (0–14.8)
80+	3542	3	1.3 (0–3.1)	0.6 (0–1.4)
**Total**	**405,636**	**143**	**37.2 (12.7–65.9)**	**39.4 (13.4–64.3)**

Disability weights of 0.21 (approximating to a limited ability to perform most activities in one of the following areas: recreation, education, procreation or occupation [24]) and 0.81 (needing assistance with instrumental activities of daily living such as meal preparation, shopping and housework [24]) were used for the early and late stage cases respectively in these calculations.

The results are expressed as mean values with 95% confidence intervals in brackets, these were obtained from 10000 Monte-Carlo simulations of the data derived from 2000–2007 Urambo sleeping sickness cases.

**Note that column totals are independently modeled and may therefore not exactly match age-specific output summaries.**

From the above YLL and YLD results, if we assume that all HAT cases were reported and no age weighting was applied then the DALY score for Urambo was 215.7 (155.3–287.5), when age weighting is applied the result increased to 281.8 (95% CI: 209.1–362.6). If 45% under-reporting and no age weighting was applied then the result was 622.5 (95% CI: 155.3–1098.9), however, when 45% under-reporting and age weighting is applied then the DALY score was 978.9 (95% CI: 201.9–1870.8). Age specific DALY scores are detailed in Table 5. If a disability weight of 0.35 is applied (25) then the DALY burden would be 573.5 (95% CI: 147.5–1002.1).

**Table 5:** Disability Adjusted Life Years (DALYs) due to *T.b.rhodesiense* sleeping sickness in Urambo, Tanzania.

Age of onset (years)	DALYs with 0% under-reporting and no age weighting	DALYs with 0% under-reporting and with age weighting	DALYs with 45% under-reporting and no age weighting	DALYs with 45% under-reporting and with age weighting
0–4	0.8 (0.7–0.8)	0.3	28.5 (0.7–75.9)	34.1 (0.3–91.2)
5–14	150.3 (147.7–154.2)	205.1 (202.2–209.5)	180.4 (147.7–218.7)	245 (202.2–295.4)
15–29	35.2 (0.2–64.4)	46.9 (0.2–85.9)	222.4 (0.2–403.2)	300.7 (0.2–547.1)
30–44	14.8 (1.8–34.0)	18.5 (2.5–43.9)	86.4 (1.8–212.0)	102.1 (2.5–249.7)
45–59	11.9 (1.6–30.5)	12.2 (1.8–30.4)	57.4 (4.3–122.1)	52.3 (4.6–108.8)
60–69	8.2 (2.1–19.6)	6.4 (1.6–14.5)	28.5 (9.6–48.6)	21.0 (7.0–35.7)
70–79	6.8 (0–24.0)	4.2 (0–14.8)	12.8 (4.1–27.5)	7.7 (2.5–16.8)
80+	1.3 (0–3.1)	0.6 (0–1.4)	3.6 (0–8.2)	1.7 (0–3.9)
**Total**	**224.4 (198.5–275.5)**	**291.4 (260.6–346.6)**	**731.7 (482.1–1067.2)**	**901.1 (603.3–1307.9)**

**Note that column totals are independently modeled and may therefore not exactly match age-specific output summaries.**

If we assume that DALY burden were estimated in the absence of AIDS pandemic, as shown in Table 6, then the DALYs with no under-reporting would have been 205.1 (95% CI: 147.5–273). When 45% under-reporting and no age weighting was applied, then the DALY burden would have increased to 585.7 (95% CI: 175–1029.8) and 761.6 (209.3 –

**Table 6:** DALYs due to *T.b.rhodesiense* sleeping sickness in Urambo assuming the absence of AIDS/HIV pandemic.

Age of onset (years)	DALYs with 0% under-reporting and no age weighting	DALYs with 0% under-reporting and with age weighting	DALYs with 45% under-reporting and no age weighting	DALYs with 45% under-reporting and with age weighting
0–4	1.2 (0–5.9)	0.5 (0–2.4)	33.8 (0–79.1)	52.4 (0–128.7)
5–14	149.4 (147.5–151.7)	204.1 (201.9–206.7)	176.8 (147.5–231.8)	194.9 (150.0–260.2)
15–29	35.9 (0–73.7)	48.9 (0–99.1)	234.6 (0–382.9)	349.9 (0–530.7)
30–44	18.4 (0–49.2)	23.1 (0–63.5)	108.1 (0–200.2)	159.8 (0–312.1)
45–59	10.5 (0–37.4)	10.7 (0–38.5)	65.7 (0–125.9)	95.9 (0–188.4)
60–69	8.0 (0–23.1)	6.2 (0–17.6)	31.3 (0–56.8)	44.2 (0–81.2)
70–79	1.4 (0–5.6)	0.8 (0–3.4)	8.9 (0–18.8)	12.9 (0–41.3)
80+	0.9 (0–3.3)	0.4 (0–1.5)	3.2 (0–7.7)	4.4 (0–12.5)
**Total**	**205.1 (147.5–273.0)**	**271.8 (205.1–349.9)**	**585.7 (17.5–1029.8)**	**805.5 (150.0–1550.1)**

### Total costs of hospitalisation for HAT patients and health services

Between January and December 2004, 143 *T. b. rhodesiense* HAT cases were parasitologically confirmed at Kaliua Health Centre, out of them 30 cases were early while the other 113 were late stage sleeping sickness cases. These patients stayed in hospital for a total of 3601 days, resulting in mean hospital stay per patient of 25 days. Following the discussion on costs above, the costs of hospital stays were estimated for all 143 patients at the conservatively low rate of TZS 2000 (US$ 2) per patient per night giving a cost of US$ 7202, and an estimated US$ 1 per patient for initial diagnosis, coming to US$ 143. Applying as the cost of the drugs to treat the disease the values estimated by WHO, 30 early stage and 113 late stage patients would cost US$ 1050 and US$ 7119 respectively to treat, Thus the total cost to the health services, including WHO, would be estimated at US$ 15,514. Of this, the patients themselves would contribute US$ 3601 (US$1 per day's hospitalisation) plus US$ 71.50 (US$0.50 admission fee per patient), making a total of US$. 3672.50 Subtracting this sum, the net cost to the health services would thus be US$ 11,841.50. These figures should be regarded as low estimates.

### Indirect costs incurred by HAT patients for the period of their stay in hospital

Apart from the costs incurred by every individual patient on admission costs, each patient required an additional of US$ 63.40 which were indirect non-medical costs to cover their travel costs, meals and accommodation for one accompanying person during their 25 day stay in hospital. Other costs such as costs incurred by health providers were not estimated in this study since Kaliua is a missionary hospital and most of the care providers are based on voluntary basis. Table 2 shows how these indirect costs were estimated for each individual patient. For the total number of 143 patients at Kaliua this would then come to US$ 9781.20.

## Discussion

Findings of the present study show that the re-emergence of HAT in Urambo district continues to impose a significant burden on health care systems as well as communities affected by the disease. The study utilized datasets from hospital records combined with estimates of under-reporting of *T. b. rhodesiense* HAT in the district. The disease is severely neglected and does not appear among the top priority diseases in any of the disease endemic districts of Tanzania. Notably, the sporadic nature of zoonotic HAT results in cases being recorded as other conditions in hospital reports, health-care seeking for HAT can be prolonged and frustrating [25] and many cases are not reported at all because the affected patients fail to reach health facilities. This study demonstrates the importance of conducting disease-specific burden assessments in local settings [10] as they provide useful epidemiological data that can be very useful in the planning, prioritisation and proper allocation of limited resources in rural areas.

The present study showed that nearly ¾ of HAT cases presented to health facilities in late stage of the disease, and almost all (98%) most presented passively. Late stage presentation has serious consequences; delays in seeking care result in reduced chance of complete cure and late presentation increases the risk of drug-associated adverse effects and the chances of treatment failure which can both result in death [26]. Late stage HAT patients suffer a much greater burden per episode and enormous stigma as the disease may be mistaken for AIDS because both diseases share clinical similarities. One explanation for late hospital presentation is that patients spent much of the early stage seeking health support from alternative sources and only after failing to recover do they decide to seek referral medical attention [25], [26]. There are no effective traditional treatments for HAT and HAT drugs are not available in any pharmacies.

Evidence shows that people often seek basic health services from more than one source, including local drug stores and occasionally traditional healers [25], [26]. These studies observed that about 20% of the communities living along the Tanzanian coast used no health services at all - an interesting finding since communities living along the coast may be considered to have a higher level of awareness on health matters and also a better health services as compared to most of the rural communities in remote areas of western Tanzania where the present study was conducted [27].

This paper presents the first assessment of the burden of *rhodesiense* HAT for Tanzania and only the second for East Africa. Some studies on the burden of other neglected diseases and zoonoses have been undertaken for Tanzania including the burden of brucellosis [28]. Its findings which are consistent with ours, both diseases show high burden that impact on local health systems as well as communities.

Estimating disease specific burdens for individual conditions can be useful, particularly when such studies use local data and can be applied at the local level. Assessments do not require much additional resource at the local level, while the results can inform local systems with regard to disease prioritisation. Improvements in record keeping should be a priority for local health facilities wishing to improve the evidence-base for resource-allocation decisions.

Previous burden of disease studies conducted in Tanzania estimated that the country loses 10 million years of life annually through illness and death. This amounts to four months of life lost per year for every man, woman and child in the country [29]. Our study also shows that families incur very substantial costs to maintain their sick family members in the health system while being treated for HAT. HAT patients require assistance with almost all activities of daily living [25], and the majority of the villages from which HAT patients arise are located far from the treatment centres. The distance to health facilities has been associated with serious delays in seeking health care services [25], [30]. It is clear that families with HAT patients will require substantial financial capacity to cover transport costs as well as maintain their patients in hospital for a prolonged duration.

The majority of affected communities are poor and have other priority health conditions to attend to (infectious diseases as well as non communicable diseases). Evidence from other rural areas of Tanzania suggests that some communities could not afford a user fee even as small as TZS 500 (approximately US$ 0.50) which represents the charge for a basic laboratory investigation [31]. The majority of families living in rural communities live below the poverty line. Studies conducted in rural districts of Tanzania during 1990–1996 suggested that 26% of rural communities were subsisting on less than US$ 1 per person per day [32]. For families in rural communities to spend such huge amount of money for diagnosis of a family member is a serious financial burden. That burden is likely to be more severe if the patient is the bread-winner, as other family members will need either to do their work on the farm or produce earnings to cover the gap caused by the sick family member - not an easy task for rural settings where employment is almost non-existent. Even if a HAT patient obtains treatment, full recovery takes several months before a patient can return to productive activities. In some cases patients may develop serious long term or even permanent complications.

Several other authors have associated household income and disease burden. A study conducted in Tanzania suggests that the majority of the rural population suffers the combined burden of diseases as most of the socio-economic activities carried at the household level does not provide sufficient resource to cover their basic health needs [33]. In Kenya, households could not afford to buy a mosquito net to protect themselves or their family members from malaria [34]. The first study of its kind to look at patients' costs for HAT [35] estimated that patients spent the equivalent 1.5 months of income on costs, rising to nearly a quarter of a year's income if their time off work were included. For HAT, in the Democratic Republic of Congo (DRC) it was found that household incomes were so limited and the costs of HAT treatment was considered to be so high that this was likely to compromise the timely receipt of medical care [35]. In DRC, patients' average financial costs came to 5 months of income and were as high as 10 months for some individuals. For families living on less than US$ 1 per day in Tanzania, the cost of US$ 68.40 of indirect non-medical costs plus US$ 25.50 of direct medical costs per patient represents a similar proportion of income.

Patients and their family members with in a household often take a long time to prepare for receiving health care and, in most cases, such preparations depend on the solidarity of family members and sometimes even community support [36]. Similarly, household members take time to ready themselves and to mobilize resources, before they seek HAT treatment on behalf of or accompany [12], [36]. In DRC it was observed that despite the communities' acceptance of active screening, the introduction of a ‘symbolic’ card fee was a major obstacle to treatment-seeking behaviour [37]. This is likely to cause very serious consequences for the household and may even compromise other family commitments such as provision of basic education to school children and even food provision to the rest of the family members.

Our study also observed that the high disease burden of HAT was largely attributed to high levels of disease under-reporting in Urambo, which suggested that for every reported case of HAT nine went un-reported [18]. Previous under-reporting studies in Uganda estimated that for every 10 reported cases of HAT, 7 went unreported, and, since untreated patients die, this meant that for every HAT death reported to health systems, twelve others went undetected [12]. Our studies also observed that HIV/AIDS pandemic may have contributed to increase the burden of HAT in Tanzania. Despite health reforms in Tanzania, expecting districts to move from managing diseases to managing health systems from an equity perspective is thought to be unrealistic [38]. The poorest societies carry the heaviest burden of diseases and it is therefore difficult for any health system to target the very poor accurately.

A recent study [32] observed that poorer families were less active and effective in seeking health care than their relatively wealthier counterparts even in rural societies that were assumed to be uniformly poor. Despite Tanzania having the higher health care seeking rates than many countries in sub-Saharan Africa, health inequalities between the very poor and wealthier families were obvious [38]. Members of better off families had higher chances of obtaining suitable treatment once ill, than did those from poor families.

There are some inherent limitations of using the decision tree model for under-reporting in Tanzania that was originally designed for Uganda. The data used to construct that model were derived from an area with better health coverage and better equipped health facilities [11] than in the area of Tanzania where this study was conducted, where most health facilities do not have even basic diagnostic equipment such as microscopes.

One of the major control challenges for HAT is the resurgence of both the gambiense and rhodesiense forms of the disease, which has been observed for rhodesiense in Tanzania as well as in many other disease endemic countries of sub-Saharan Africa. Some of the recent examples of these resurgences have been reported in several countries [14], [15], [39], [40], and there have also been reports of the occurrence of new foci of HAT in many areas of sub-Saharan Africa including Uganda and Tanzania [41], [42]. This resurgence was linked to a lack of adequate knowledge regarding disease trends as well as of the control options in many of the affected communities as well among some policy makers [7], [43]. This in turn was linked to the lack of sufficient resources to sustain regular surveillance activities. This stresses the urgent need for disease specific burden estimation in such communities, as it will allow proper allocation of the limited available resources in poor communities. Zoonotic HAT affects both rural communities as well as the animals. Previous studies observed that the lowest level of effective health seeking behaviour was observed among livestock keepers [30]. Control needs to be targeted at both humans and animals and needs to be a shared responsibility between medical and veterinary sectors [44].

Being one of the most neglected diseases, in most cases HAT is not considered an important disease in most of the affected communities as there is a tendency for policy makers to rank the disease according to their importance in the communities simply by looking at national mortality figures. Findings from our study demonstrated that HAT consumes a very significant proportion of workforce resource, time and hospital space. Also the results of this study demonstrated the importance of conducting disease-specific burden studies, particularly in rural settings rather than generalizing using regional or national figures, reinforcing the conclusions of earlier work [10]. There is a need to target the limited available resources more efficiently so as prevent future outbreaks. In deciding areas requiring prioritisation it is important to use estimates from community perspectives. Local burden of disease estimates are important aspects as they provide good epidemiological data which can be used for timely planning and proper resource allocation in most of the local health care settings and inform disease prioritisation in rural settings.
